# *HSPA13* gene and microRNA-155: relationship between Down syndrome and Alzheimer’s disease

**DOI:** 10.1590/1980-5764-DN-2024-0239

**Published:** 2025-09-05

**Authors:** Fabiana de Campos Gomes, Beatriz Pavarino Bertelli, Conceição Pinheiro de Souza, Daniel Ramos de Oliveira Santos, João Simão de Melo-Neto, Érika Cristina Pavarino, Eny Maria Goloni-Bertollo

**Affiliations:** 1 Faculdade de Medicina Ceres, São José do Rio Preto SP, Brazil.; 2 Faculdade de Medicina União dos Lagos, São José do Rio Preto SP, Brazil.; 3 Faculdade de Medicina de São José do Rio Preto, Departamento de Biologia Molecular, São José do Rio Preto SP, Brazil.; 4 Universidade Federal do Pará, Faculdade de Terapia Ocupacional e Fisioterapia, Instituto Ciências da Saúde, Belém PA, Brazil.

**Keywords:** Heat-Shock Proteins, MicroRNAs, Down Syndrome, Alzheimer Disease, Proteínas de Choque Térmico, MicroRNAs, Síndrome de Down, Doença de Alzheimer

## Abstract

Gene dysregulation in trisomy 21 can cause disorders of genes that are members of the heat-shock proteins (HSPs) family and contribute to the early onset of Alzheimer’s disease (AD) in Down syndrome (DS).

**Objective:**

Investigate *in silico* differently expressed genes (DEGs) of HSPs and the interaction with microRNAs (miRNAs) located in human chromosome 21 (Hsa21).

**Methods:**

Two transcriptome libraries of human brain samples, datasets GSE5390 (DS) and GSE33000 (DA), were extracted from the Gene Expression Omnibus (GEO) and analyzed via GEO2R. DEGs with p-values (Adj p-values) <0.05 were analyzed via STRING. MiRNAs were identified in the miRbase database and analysis of their potential regulation on DEGs was performed using the DIANA tools.

**Results:**

*HSPE1*, *HSP90B1*, *HSPB8* and *HSPA13* genes showed a different expression pattern in the transcriptomes of DS. The *HSPA13* and *HSPA2* genes showed an altered expression profile in the DS and AD datasets. In the predicted protein-protein interactions (PPI), we identified the interaction of HSPE1, HSP90B1, HSPB8 and HSPA13 with other HSP proteins. The miRNA encoded by Hsa21 (hsa-miR-155-5p) interacted with the *HSPA13* gene.

**Conclusion.:**

The results suggest that certain genes encoding members of the *HSP* family, and in particular the interaction between miR-155-5p and *HSPA13*, may be associated with AD in DS.

## RESUMO

A desregulação de genes na trissomia 21 pode afetar a expressão de genes que são membros da família das proteínas de choque térmico (HSPs, do inglês *heat shock proteins*) e contribuir para o a surgimento precoce da doença de Alzheimer (DA) em indivíduos com síndrome de Down (SD).

**Objetivo:**

Investigar *in silico* os genes diferencialmente expressos (GDE) que codificam proteínas de choque térmico e suas interações com microRNA (miRNA) localizados no cromossomo 21 humano (Hsa21).

**Métodos:**

Duas bibliotecas de transcriptomas de amostras de cérebros humanos, os conjuntos de dados GSE5390 (DS) e GSE33000 (DA), foram extraídos do Gene Expression Omnibus (GEO) e analisados pelo GEO2R. Os GDE com valores de P (Adj p-values) <0,05 foram analisados via STRING. miRNA foram identificados no banco de dados miRbase e a análise de seu potencial em regular GDE foi realizada usando as ferramentas DIANA.

**Resultados:**

Os genes *HSPE1*, *HSP90B1*, *HSPB8* e *HSPA13* mostraram um padrão de expressão diferente nos transcriptomas de DS. Os genes *HSPA13* e *HSPA2* mostraram um perfil de expressão alterado nos conjuntos de dados DS e AD. Nas interações proteína-proteína (PPI) previstas, identificamos a interação de HSPE1, HSP90B1, HSPB8 e HSPA13 com outras proteínas de HSP. O miRNA codificado por Hsa21 (hsa-miR-155-5p) interagiu com o gene *HSPA13*.

**Conclusão:**

Os resultados sugerem que certos genes que codificam membros da família *HSP*, particularmente a interação entre miR-155-5p e *HSPA13*, podem estar associados à DA na SD.

## INTRODUCTION

 Down syndrome (DS), also known as trisomy 21 (T21), is a chromosomal abnormality caused by autosomal aneuploidy on human chromosome 21 (HSA21). An imbalance in gene dosage affects the expression and regulation of genes located on HSA21 and other chromosomes, resulting in a genome-wide impact with various implications for transcriptional control^1,2^ and the coding of microRNAs (miRNAs)^3,4^. 

 Dysregulation of gene and miRNA expression has a direct effect on the phenotypic spectrum of the syndrome, resulting in variations in clinical signs, physiological aspects, and morphofunctional characteristics across multiple body systems, such as the central nervous system (CNS)^1,5^. In the CNS, alterations in the number and morphology of neuronal cells, cytoarchitectonic disorganization of the cerebral cortex, and reduced volume of the frontal and parietal lobes have been described^6,7^. In addition to intrinsic abnormalities in the CNS, individuals with DS are prone to developing neuropathology, such as early-onset Alzheimer’s disease (AD)^1,6-8^. In DS, the extra copy of amyloid beta precursor protein (APP) located on HSA21 is linked to the early onset of dementia^1,5^. 

 Increased *APP* expression and abnormalities in APP cleavage result in the abnormal production of 40- and 42-amino-acid beta-amyloid (βA) peptides (βA-40 and βA-42)^8-11^. The βA-40 and βA-42 isoforms are considered pathogenic amyloidogenic peptides with a greater propensity to aggregate, accumulate, and deposit in nervous tissue^9,10,12^. MiRNAs have direct implications in the neurobiological processes associated with the pathophysiology of AD^13-15^. These molecules interfere with the stability and translation mechanisms of messenger RNA (mRNA) and various cellular and molecular pathways^16^. In this context, understanding the expression profile of genes involved in the proteostasis network, as well as the interaction of these genes with miRNAs encoded on HSA21, may help in understanding the mechanisms related to the onset of AD in DS. 

 Among the genes integrated into the proteostasis network, molecular chaperones from the heat shock protein (*HSP*) family play important biological roles in various cellular processes, such as disaggregation, degradation of misfolded proteins, and reduction of toxic oligomers such as beta-amyloid peptides and hyperphosphorylated Tau^17,18^. In DS, the number of studies related to the effects of trisomy 21 on the proteostasis network is limited^19-21^; thus, incorporating information about the expression profile of transcripts from *HSP* family members and their interaction with miRNAs in brain tissue samples from DS individuals may help in understanding the effects of trisomy 21 on the proteostasis network. In the present study, we identified differentially expressed genes (DEGs) encoding HSPs in the brain transcriptomes of adult individuals with DS. We subsequently analyzed the signaling pathways at the gene-protein interface and the interactions of miRNAs with the DEGs of *HSPs* to identify potential biomarkers that could be used to better understand AD in DS. 

## METHODS

### Data collection and processing

 The search for the transcript dataset was conducted in the GEO DataSets database deposited in the Gene Expression Omnibus (GEO) repository (https://www.ncbi.nlm.nih.gov/gds) of the National Center for Biotechnology Information (NCBI). 

 To identify the dataset, we used the following filters: (("Down syndrome"[All Fields] AND "brain"[All Fields]) AND "*Homo sapiens*"[porgn]) AND ("gse"[Filter] AND "Expression profiling by array"[Filter] AND ("2007/01/01"[PDAT]: "2021/12/31"[PDAT])) and (("Alzheimer disease"[All Fields] AND "brain"[All Fields]) AND "*Homo sapiens*"[porgn]) AND ("gse"[Filter] AND "Expression profiling by array"[Filter] AND ("2007/01/01"[PDAT]: "2021/12/31"[PDAT])). Among the identified series, we considered the array datasets from the series GSE5390 - ID: 2941 (trisomy 21 transcriptome) and GSE33000 - ID: 200033000 (Alzheimer’s disease). 

### Sample information

The GSE5390 series is an expression profile based on the GPL96 platform (Affymetrix Human Genome U133A Array) and contains brain tissue samples from the dorsolateral and prefrontal cortex of adult humans (aged 47–63 years) with DS (seven samples) and healthy controls (eight samples). The GSE33000 series, an expression profile based on the GPL4372 platform (Rosetta/Merck Human 44k 1.1 microarray), contains prefrontal cortex samples collected postmortem from individuals with Alzheimer’s and Huntington’s diseases (624 samples), from which we selected only the DA data (310 samples) and nondementia control data (157 samples).

### Selection and analysis of heat shock protein-encoding differently expressed genes

 In the raw dataset spreadsheets of the GSE5390 (DS) and GSE33000 (AD) series, all the HSP family genes with adjusted p values (Adj p values) <0.05 were selected via the "find and select" tool in Microsoft Excel®. Upon identifying the genes with an Adj p value <0.05, data related to the ID, p value, moderated t statistic, B statistic, logFC (log2-fold change), gene symbol, and gene title were collected. All the collected data were transferred to a new spreadsheet for further analysis. 

### Analysis of predicted protein-protein interactions (PPIs)

 The construction and extraction of PPI information were performed via the STRING database (https://string-db.org/) version 12.0. The enrichment settings for mapping were configured according to the following settings: text mining, experiments, database with a high-confidence interaction score (0.900), and a maximum of five interactions. 

### Identification of microRNAs and their associations with differently expressed genes

 The miRBase data platform (https://www.mirbase.org/) was used to identify miRNAs. The functional investigation of miRNAs associated with the expression of DEGs was performed using DIANA tools server (https://diana.e-ce.uth.gr/tools). 

### Data analysis

 During the processing and analysis of the datasets collected from the GEO database, we used the GEO2R tool (www.ncbi.nlm.nih.gov/geo/geo2r). In the first analysis, we compared GSE5390 samples versus controls; in the second analysis, we selected and compared AD samples from the GSE33000 dataset versus controls. For the GEO2R analyses, we applied the following options: adjust the p values via the Benjamini & Hochberg false discovery rate method with a cutoff <0.05. For the comparison analysis, intersection calculation, and visualization of upregulated and downregulated genes, we used the Venn diagram tool available on the Bioinformatics & Evolutionary Genomics portal (https://bioinformatics.psb.ugent.be/webtoolsR/Venn/). 

 To estimate the proportion of false-positive genes with differential expression for HSPs, we used the false discovery rate (FDR) online calculator (https://tools.carbocation.com/FDR). From the information obtained from the FDR and LogFC, we identified upregulated and downregulated genes. Genes with positive fold change values were considered to have increased gene expression, and those with negative fold change values were considered to have decreased gene expression, as described by Mutch et al.,^22^. Genes that exceeded the cutoff value (−log10 p value=2.0) were considered to have high statistical significance^23^. 

 For the protein interaction analyses, we used the significance level indicated by the PPI enrichment p value provided by the STRING database. With respect to the enrichment analysis and functional annotation of the miRNAs, we considered a high interaction score >0.8 obtained via DIANA tools (https://dianalab.e-ce.uth.gr/home). 

## RESULTS

### Identification of differently expressed genes and the heat shock proteins expression profile

**Figure 1:**
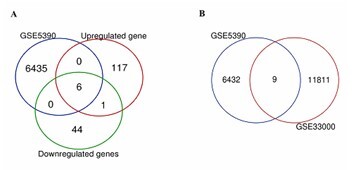
Screening and analysis of differently expressed genes (DEGs). (A) DEGs total (blue), upregulated (red) and downregulated (green) genes. (B) Intersection analysis of the GSE5390 and GSE33000 microarray datasets.

 The analysis conducted via the Venn diagram tool revealed upregulated gene expression and downregulated gene expression in the transcript samples from GSE5390 (DS) (Figure 1A). In the intersection analysis, we identified the sharing of nine genes between the GSE5390 (DS) and GSE33000 (AD) datasets (Figure 1B). 

**Figure 2:**
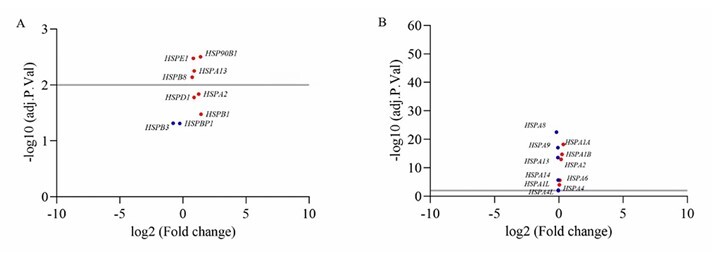
RNA-seq volcano plot of differently expressed genes (DEGs) from HSP family members. (A) Upregulated and downregulated genes in GSE5390 (DS). (B) Upregulated and downregulated genes in GSE33000 (AD). Red dots: upregulated genes. Blue dots: downregulated genes. Light gray line: significance threshold. The –log10 (p values=2) represents the level of significance. The log2-fold change represents the difference in the expression level of each gene between the SD and control groups and between the DA and control groups.

 When the *HSP* genes from the GSE5390 (DS) series were compared with those from the GSE33000 (AD) series, only the *HSPA13* and *HSPA2* genes showed altered expression profiles in both data sets (DS and AD). In particular, overexpression of *HSPA13* was observed in DS (Figure 2A and 2B). 

 In GSE5390 (DS), nine *HSPs* genes presented an altered expression profile, of which seven were upregulated (*HSPE1, HSPB8, HSPA2, HSP90B1, HSPA13, HSPD1*, and *HSPB1*) and two were downregulated (*HSPB3* and *HSPBP1*). After FDR analysis, only four genes, including *HSPE1, HSP90B1, HSPB8* (encoded on chromosome 12) and *HSPA13* (encoded on chromosome 21), were significantly different (Figure 2A). 

### Heat shock proteins exhibit functional interactions with proteins involved in protein folding and clearance of misfolded and aggregated proteins

**Figure 3:**
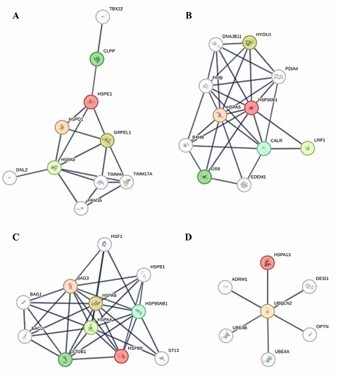
Protein-protein interactions from HSP are differentially expressed. First shell of interaction from HSPs (red circle line). (A) HSPE1. (B) HSP90B1. (C) HSPB8. (D) HSPA13. Analysis was performed with STRING (PPI enrichment p<0,05; confidence score 0.900).

 We investigated the protein-protein interactions (PPIs) of the HSPs to identify the PPIs with the highest confidence score, 0.900. The results for the four genes (*HSPE1, HSP90B1, HSPB8*, and *HSPA13*) that were significant in the FDR analysis are shown in Figure 3. In the first set of interactions in the PPI network, we identified interactions of HSPE1 with GRPEL1 and HSPD1. For HSP90B1, interactions with HSPA5, HYOU1, and LRP1 were observed. HSPB8 interacts in the first shell with HSPA8, HSPA4, and HSP90AB1. For HSPA13, we identified a first-shell interaction with UBQLN2. 

### Prediction analysis of interactions between microRNAs and differently expressed genes

 The miRNA hsa-miR-155-5p, encoded on chromosome 21, was the only miRNA that interacted with the *HSPA13* gene (score 0.451). A more comprehensive analysis involving miRNAs, independent of chromosomal location, revealed an interaction between the nine DEGs and 53 miRNAs, of which 12 had a score >0.8, with three HSPs differentially expressed in the DS transcriptome, including *HSPD1, HSPB8*, and *HSPA13*. However, to refine the present study, we focused on the interactions of miRNAs with the *HSPA13* and *HSPB8* gene because of the statistical significance after FDR application. After analysis, we identified the interactions of eight miRNAs (hsa-miR-3607-5p, hsa-miR-200a-3p, hsa-miR-141-3p, hsa-miR-374b-5p, hsa-miR-200b-3p, hsa-miR-1277-5p, hsa-miR-200c-3p, and hsa-miR-33a-3p) with the *HSPA13* and four miRNAs (hsa- miR-218-1-3p e hsa-miR-425-5p) with *HSPB8*. 

## DISCUSSION

 In DS, an imbalance in the expression of genes located on chromosome 21 affects gene expression patterns and miRNA coding^7,3,24^. These dysregulations impact the entire genome, resulting in increased susceptibility to diseases such as AD^7^. In this context, understanding the expression patterns of *HSP* genes encoding chaperones involved in certain biological mechanisms, such as the degradation of misfolded proteins or amyloidogenic aggregates^25^, can help elucidate the mechanisms related to AD in DS. In this study, we investigated the expression profile of transcripts from the *HSP* family in brain tissue samples from the prefrontal cortex of adults with DS and compared them with AD transcripts. Additionally, we analyzed the protein-protein interaction network and miRNA integration with the differentially expressed *HSPs*. 

 In the transcriptome of GSE5390 (DS) derived from prefrontal cortex brain tissue samples, we identified alterations in the expression patterns of several genes, including *HSP* transcripts. This brain region plays an essential role in planning, cognitive coordination, and behavior^26^. Studies have shown that the prefrontal cortex is affected by AD and is a brain region prone to the deposition of β-amyloid aggregates^27^, making it a good tissue model for investigating neuropathological mechanisms. 

 In transcriptome samples from the prefrontal cortex of DS and AD patients, we observed distinct and peculiar changes in the expression patterns of certain genes. This could be hypothesized for two reasons:

- Lack of evidence regarding the presence or absence of dementia, as per the available information in the GEO datasets for the GSE5390 series;
- Lack of information regarding similar or distinct molecular mechanisms concerning neuropathology between AD and AD in DS.

In fact, even when we are dealing with assumptions, in this study, when we compared the DEGs between AD patients and DS patients, we identified an increase in *HSPA2* expression in both the AD and DS transcriptomes and an increase in *HSPA13* expression restricted to DS. Recent studies with bioinformatics data and mutant *APP* cell lines have identified the *HSPA2* gene as a potential molecular biomarker for the prognosis of late-onset AD^28^. This information is based on certain findings, such as the increase in *HSPA2* expression in parallel with the increase in βA40 and βA42 peptide levels in mutant *APP* cell lines and neuroglioma cell lines^28,29^. Considering these previous findings and information about the function of HSPA2, which includes the ability to prevent protein aggregation^18^, the upregulation of *HSPA2* in DS (GSE5390), similar to that in AD (GSE53000), could be a target gene for investigating potential signs of dementia. 

 Regarding the particularities of *HSP* expression abnormalities in DS, after analyzing the FDR values, we identified increases in *HSPE1*, *HSP90B1*, *HSPB8*, and *HSPA13*. The *HSP* family encodes HSP proteins whose molecular weight, functional role, and tissue localization differ^18,30^. HSPs work in conjunction with the ubiquitin-proteasome pathway, playing crucial roles in various cellular mechanisms, including the folding of newly synthesized polypeptides and the prevention and degradation of misfolded proteins^18^. 

 In the brain, among the functions of the differentially expressed HSPs in DS identified in this study, we found variations in the functions and cellular processes involved, according to the literature. Previous findings have shown that *HSPE1* functions as a cochaperone of *HSPD1*, assisting in mitochondrial protein folding and is associated with neurodegeneration^31,32^; *HSP90* interacts with protein aggregates and is involved in the clearance of β-amyloid^31,33^; and *HSPB8* contributes to neuroprotection and may be expressed in reactive astrocytes near senile plaques^31,34^; however, studies on the functional role of *HSPA13* in the context of neurodegeneration are lacking. In a recent study, Gao et al.^35^ identified *HSPA13* as a new target for studies of the TNFα signaling pathway in response to cell death. In light of these findings, we encourage further investigation into *HSPs* in DS, as they are considered "professional chaperones" because of their role in preventing misfolded proteins and, consequently, protein aggregation^36^. 

 Interestingly, the genes *HSPE1*, *HSP90B1*, and *HSPB8* are encoded on chromosome 12, and *HSPA13* is encoded on chromosome 21, specifically in the chromosomal region reported to be affected by early-onset Alzheimer’s disease (EOAD)^10^. This information not only reveals variations in the expression patterns of specific *HSPs* but also raises questions about the impact of trisomy 21 on specific chromosomes, particularly in the case of chaperone-encoding genes. 

 Notably, when we evaluated the PPI interaction network, we identified that *HSPPE1*, *HSP90B1*, *HSPA8*, and *HSPA13* strongly interact with specific proteins, including other members of *HSPs* and proteins, which, according to information obtained from the protein database (NCBI [https://www.ncbi.nlm.nih.gov/protein/]), function in specific roles, including in general, the folding and degradation of protein aggregates. Through PPI analysis, it is possible to understand the protein complexes that control biological activities. In the cell, PPIs are referred to as the "interactome" and play important roles in physiological and pathological processes^37^. Thus, as observed in this study, interactions of HSPs with elevated levels of specific proteins may lead to possible implications for cellular homeostasis since an increase in gene expression affects protein expression, creating an imbalance in numerous processes, such as the modulation of signaling pathways, protein localization, protein folding, protein degradation, and the formation of abnormal protein complexes, which can culminate in cellular toxicity^38^. 

 With respect to the findings of miRNA interactions with , to the best of our knowledge, no studies have established a relationship between miRNAs and *HSP* genes in DS. In this study, the weak interaction of miRNA-155 with the *HSPA13* gene (score 0.451) observed in our *in silico* analysis may not necessarily prevent its biological action *in vivo*. The literature describes a direct and indirect contribution of this miRNA to the onset or progression of DA, as it can simultaneously reduce the translation of several genes at the posttranscriptional level^4,39^. 

 Although the role of miRNAs in gene expression regulation through nuclear and cytoplasmic mechanisms is well established^40,41^, in the present study, owing to the *in silico* design, it was not possible to evaluate the regulatory mechanisms of miRNAs with *HSPA13*. In addition to methodological limitations, we need to overcome the challenges of reproducing conditions closer to those observed in DS, as there are extra copies of genes and a lack of knowledge regarding the impacts of this genetic imbalance on miRNA-target regulatory mechanisms. In a preliminary literature review, we believe that hsa-miR200a-3p, identified here as a target of *HSPA13*, could be a good starting point for understanding possible mechanisms involving miRNAs and chaperones. This hypothesis is based on studies that evaluated the role of hsa-miR-200a-3p in regulating specific genes involved in the control of beta-amyloid and hyperphosphorylated tau production, thus highlighting this miRNA as a potential plasma biomarker for detecting AD^42,43^. 

 In summary, in the DS transcriptome, we detected alterations in the expression patterns of certain *HSPs*. For protein-protein interactions, discovering the protein targets that interact with *HSPs* opens new paths to elucidate possible implications in cellular interactomes. In parallel, the exposure of interactions between miRNAs and *HSPA13*, as shown in this research, may help in new discoveries related to miRNA-mediated control mechanisms in response to the increased expression of their target genes and, consequently, the possible repercussions on the gene-protein interface. Finally, although the role of *HSPs* in the health-disease process is well established^18^, investigations into the identification of *HSPs* and their respective encoded proteins, as well as the miRNAs that interact with *HSP* target genes, can contribute to advancing research related to understanding AD in DS. 

 Although the results of our study are novel, some limitations should be noted, such as the small number of *in silico* samples, the standardization of a cutoff threshold for log2 (fold change) to select DEGs, and the lack of epidemiological data on individuals with DS, such as data on comorbidities, including dementia. These limitations may restrict the interpretation of the biological response in future *in vivo* or *in vitro* analyses. For this reason, it is necessary to consider the need to evaluate molecular mechanisms through different experimental models and specific targets, as certain *HSP* genes play specific roles in a given biological process^18^. Finally, in general terms, in this research, we added a "new brick in the wall," which we hope will help lay the foundation for understanding and identifying biomarkers to aid in the screening, diagnosis, and treatment of AD in DS. 

## Data Availability

The datasets generated and/or analyzed during the current study are publicly available at Gene Expression Omnibus (GEO) repository (https://www.ncbi.nlm.nih.gov/gds) of the National Center for Biotechnology Information (NCBI).
